# Significance of plasma TGF-β1 level detection in patients with T2DM with heart failure

**DOI:** 10.5937/jomb0-47321

**Published:** 2024-09-06

**Authors:** Yunjing Sun, Bo Miao, Yabing Cao, Jiangman Cui, Yingxiao Da, Liping Qi, Song Zhou

**Affiliations:** 1 Xingtai Third Hospital, Cardiology Ward 2, Xingtai, China; 2 Xingtai Third Hospital, Coronary Care Unit, Xingtai, China; 3 Xingtai Third Hospital, Cardiology Ward 1, Xingtai, China; 4 Xingtai Third Hospital, Department of Cardiology, Xingtai, China

**Keywords:** heart failure, NT-proBNP, type 2 diabetes mellitus, TGF-b1, srčana insuficijencija, NT-proBNP, dijabetes melitus tip 2, TGF-b1

## Abstract

**Background:**

The aim of the study was to examine the significance of plasma Transforming Growth Factor-1/TGF-β1 (TGF-β1) level testing in patients with Type 2 Diabetes Mellitus (T2DM) and heart failure.

**Methods:**

A sample of T2DM patients who were hospitalised for dyspnea was chosen between June 2021 and June 2023. Based on the convenience sample approach, 150 cases were screened for the study, and 50 healthy non-diabetic people without cardiac problems who completed physical examinations over the same period were included as a control group. All study participants had their serum NT-proBNP and plasma TGF-I levels checked, and the values between the two groups were compared. Then, the patients with T2DM with heart failure were grouped according to whether they were accompanied by heart failure or not and the grading of cardiac function, and then the serum NT-proBNP and plasma TGF-β1 levels were compared between the different groups of patients. The diagnostic value of plasma TGF-β1 in the occurrence of heart failure in patients with T2DM was analysed.

**Results:**

There were 54 patients without heart failure and 96 people with heart failure among the 150 T2DM patients. The cut-off point was 44.50 g/L. At this time, the sensitivity and specificity for diagnosing concomitant heart failure in T2DM were 79.63% and 52.51%, respectively. 96 individuals with T2DM and heart failure showed greater serum and plasma levels of NT-proBNP and TGF-β1 compared to the other two groups (P=0.05). ProBNP and plasma TGF-β1 levels had a positive and significant relationship (P=0.05).

**Conclusions:**

Plasma TGF-β1 levels were much higher in T2DM patients than in the general population, and the increase in this index was more pronounced in patients who also had heart failure, a diagnostic indicator for T2DM and heart failure.

## Abstract (sr)

**Uvod:**

Cilj ovog istraživanja je bio da se ispita značaj testiranja nivoa plazme Transformišućeg faktora rasta-1/TGFb1 (TGF-b1) kod pacijenata sa dijabetesom melitusom tip 2 (T2DM) i srčanom insuficijencijom.

**Metode:**

Odabran je uzorak pacijenata sa T2DM koji su hospitalizovani zbog dispneje između juna 2021. i juna 2023. godine. Na bazi uzorka pogodnosti, pregledano je 150 slučajeva za studiju, a 50 zdravih osoba koje nisu dijabetičari i koje nemaju srčane probleme, a koje su završile fizikalne preglede u istom periodu, je uključeno u studiju kao kontrolna grupa. Svim učesnicima studije provereni su nivoi serumskog NT-proBNP i plazme TGF-I, a vrednosti između dve grupe su upoređene. Zatim su pacijenti sa T2DM i srčanom insuficijencijom grupisani prema tome da li su imali srčanu insuficijenciju ili ne, kao i prema stepenu srčane funkcije, i zatim su nivoi serumskog NT-proBNP i plazme TGF-b1 upoređeni između različitih grupa pacijenata. Analizirana je dijagnostička vrednost plazme TGF-b1 u pojavi srčane insuficijencije kod pacijenata sa T2DM.

**Rezultati:**

Među 150 pacijenata sa T2DM, 54 pacijenta nisu imala srčanu insuficijenciju, dok je 96 osoba imalo srčanu insuficijenciju. Granična vrednost bila je 44,50 g/L. U ovom trenutku, senzitivnost i specifičnost za dijagnostikovanje srčane insuficijencije uz T2DM su bile 79,63% i 52,51%, respektivno. 96 osoba sa T2DM i srčanom insuficijencijom pokazalo je veće serumske i plazmatske nivoe NT-proBNP i TGF-b1 u poređenju sa ostale dve grupe (P=0,05). Nivoi ProBNP i plazmatskog TGF-b1 su imali pozitivnu i značajnu korelaciju (P=0,05).

**Zaključak:**

Nivoi plazme TGF-b1 su bili znatno viši kod pacijenata sa T2DM nego u opštoj populaciji, a porast ovog indeksa je bio izraženiji kod pacijenata koji su takođe imali srčanu insuficijenciju, što predstavlja dijagnostički pokazatelj za T2DM i srčanu insuficijenciju.

## Introduction

Heart failure (HF) is a complicated clinical syndrome brought on by abnormal ventricular systolic function. It occurs at the end stage of the progression of all types of heart disease, with dyspnea as the primary clinical symptom. Morbidity, readmission, and mortality rates are high, and the 5-year survival rate is similar to that of patients with malignant tumours, which is around 50%. As such, it has emerged as a significant risk factor for human health [1] [2] [3] [4]. The HF Section of the European Heart Association noted in 2018 that Type 2 diabetes mellitus (T2DM) is a significant risk factor for the development of HF and that it is the cause of HF in about 30% to 40% of patients [5] [6]. Patients with T2DM are also 2 to 4 times more likely to develop HF than those without the condition. The clinical symptoms, cardiac function, and even quality of life of T2DM patients are all adversely affected by complicated HF, and the mortality risk is increased by 10–12 times [5]. Therefore, reducing the risk of HF in T2DM patients has always been a key direction of clinical research, and there are more clinical studies about risk prediction model analysis at home and abroad, but how to quickly detect and diagnose the occurrence of HF is also one of the key research contents while preventing the risk of HF for T2DM patients. Transforming growth factor-β1 (TGF-β1), one of the pro-fibrotic cytokines, promotes the onset of tissue fibrosis and assumes a crucial role in the physiological changes of myocardial fibrosis, triggering the accumulation of Periostin protein, which reduces the adhesion between cardiomyocytes and myocardial fibroblasts and induces cardiac dilatation, resulting in the development of HF [7] [8] [9]. Based on the above theory, TGF-β1 was selected as an evaluation index in this study to analyse its diagnostic value in T2DM with HF, and the results are as follows:

## Materials and methods

### Research object

Sampling was done among T2DM patients treated at the hospital from June 2021 to June 2023 due to dyspnea, and 150 cases were screened for the study based on the convenience sampling method as the study group. Inclusion criteria: (1) aged 41 years; (2) satisfied the Chinese Guidelines for the Prevention and Treatment of T2DM (2017 edition) [10] diagnostic criteria for T2DM, with a disease duration of more than two years; (3) all complained of the presence of dyspnoea symptoms and suspected HF at the time of admission; (4) had complete clinical data; and (5) signed a written informed consent form for this study. Exclusion Criteria: (1) concomitant renal impairment, acute and chronic inflammation, and tumours; (2) type 1 diabetics; (3) admitted to hospital for acute myocardial infarction; (4) treated with cardiopulmonary resuscitation (CPR) after admission to the hospital; and (5) respiratory distress due to chest trauma. The control group comprised 50 healthy persons who had undergone physical examinations over the same period, were not diabetic, and showed no abnormalities in heart function. The study group included 99 males and 51 females. There were 21 girls and 29 males in the control group, all of whom were 61.78 10.08 years old.

### Research methods

Blood samples: Before starting medication, 5 mL of fasting venous blood was drawn from patients. It was centrifuged into two parts – one with anticoagulant and the other without. The supernatant was then obtained to determine the concentrations of 1 NT-proBNP and TGF-β1 in the serum and plasma, respectively.

Diagnostic criteria for HF: The diagnostic criteria for HF from the Chinese Guidelines for Diagnosis and Treatment of HF 2018 [11] were used to assess HF: the presence of HF symptoms, a lowered or normal LVEF, but imaging-detected diastolic dysfunction of the heart, and serum concentrations of NT-proBNP (brain natriuretic peptide precursor) >300 pg/mL.

Grouping: According to the existence or absence of HF, T2DM patients were divided into groups, and the serum NT-proBNP and plasma TGF-β1 levels were compared. Patients were also classified according to the Functional Classification of the New York Heart Association (NYHA) [12]. The serum NTproBNP and plasma TGF-β1 levels of patients with various cardiac function classes were then compared. Patients with T2DM with HF were then categorised according to cardiac function class.

### Statistical analysis

The data were statistically analysed using SPSS22.0. The effect of plasma TGF-β1 levels on T2DM patients with HF was analysed using the ROC curve. In T2DM patients with HF, the relationship between plasma TGF-β1 level and serum NT-proBNP was investigated by Pearson, and the diagnostic value of plasma TGF-β1 level in T2DM patients with concurrent HF was investigated using the ROC curve.

## Results

Patients with and without HF and HF+T2DM were compared for plasma TGF-β1 levels.

**Figure 1:**
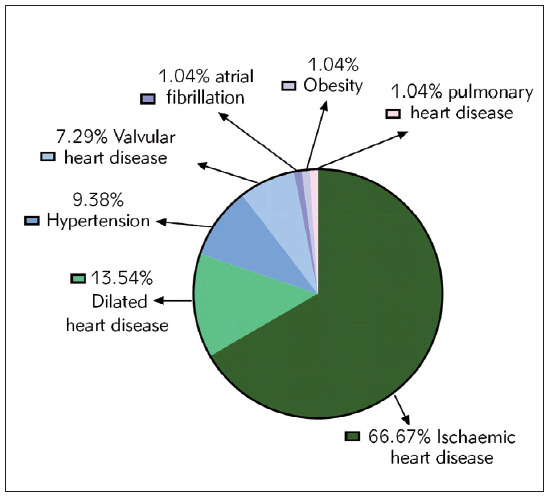
Etiological composition of HF in patients with T2DM with HF.

Among the 150 patients with T2DM, there were 96 patients with HF and 54 patients without HF. Among the 96 patients with T2DM with HF, the causes of HF were 64 cases of ischemic heart disease, 13 cases of dilated cardiomyopathy, 9 cases of hypertension, 7 cases of valvular heart disease, 1 of atrial fibrillation, 1 of obesity, and 1 of pulmonary heart disease, respectively, as shown in Figure 1.

### Plasma TGF-β1 levels in T2DM with HF: diagnostic value

**Figure 2:**
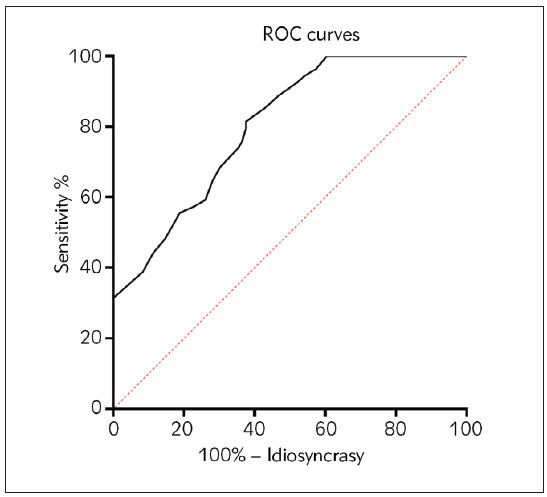
ROC of plasma TGF-b1 levels for the diagnosis of T2DM with HF.

ROC curve analysis of 96 patients with T2DM with HF compared with 54 patients with T2DM without HF within the Research group showed that its cutoff point was 44.50 µg/L, at which time its sensitivity and specificity for diagnosing patients with T2DM. The sensitivity and specificity of concurrent HF were 79.63% and 52.51%, respectively. For details, see Figure 2 and Table 1 Table 2.

**Table 1:** Comparison of serum NT-proBNP and plasma TGF-β1 levels in different groups of study subjects.

Group		number of examples	NT-proBNP (pg/mL)	TGF-β1 (mg/L)
Research group	With HF	96	1050.82±369.42*#	49.18±11.89*#
	Without HF	54	196.63±50.20*	35.70±9.11*
	**Total**	**150**	725.69±496.40*	44.33±12.72*
Control group		50	80.56±14.08	30.50±8.68

**Table 2:** Predictive efficacy of serum Gal-3 levels in the diagnosis of heart failure.

Projects	AUC	P	95%CI	cut-off point (mg/L)	Sensitivity (%)	Idiosyncrasy (%)
TGF-β1	0.803	<0.0001	0.734～0.871	44.50	79.63	52.51

### Comparison of plasma TGF-β1 and serum NT-proBNP levels in T2DM patients with HF with different cardiac functions

31 individuals had NYHA cardiac function Level II, 40 had Level III, and 25 had Level IV among the 96 patients with T2DM with HF. As shown in Table 3, serum NT-proBNP and plasma TGF-β1 levels were higher in patients with Level III cardiac function compared to those with Level II cardiac function. Table 4

**Table 3:** Comparison of plasma TGF-β1 and serum NT-proBNP levels in individuals with T2DM who have HF with various cardiac functions (±s, score).

NYHA<br>Cardiac<br>function<br>classification	Number<br>of<br>examples	NT-proBNP<br>(pg/mL)	TGF-β1<br>(mg/L)
Level	31	701.32±219.62^ab^	37.65±7.77^ab^
Level	40	1010.40±377.63^b^	49.95±7.86^b^
Level	25	1443.12±192.04	62.24±5.75

**Table 4:** Patients with T2DM and HF who had their plasma TGF-β1 levels and serum NT-proBNP levels examined.

variant	NT-proBNP	
	r	P
TGF-β1	0.784	<0.0001

### Analysis of the relationship between plasma TGF-β1 and serum NT-proBNP in patients with T2DM and HF

Comparing the levels of plasma TGF-β1 and serum NT-proBNP in people with T2DM who had HF and different cardiac functions.

## Discussion

The study revealed significant findings regarding the association between heart failure and type 2 diabetes mellitus (T2DM) among the study participants. Using a cut-off point of 44.50 g/L, the sensitivity and specificity for diagnosing heart failure in T2DM patients were found to be 79.63% and 52.51%, respectively. Furthermore, individuals with both T2DM and heart failure exhibited notably elevated serum and plasma levels of NT-proBNP and TGF-β1 compared to those without heart failure. Additionally, a positive and significant correlation was observed between proBNP levels and plasma TGF-β1 levels, indicating a potential interplay between these biomarkers in the context of heart failure and T2DM.

Additionally, our study explored the association between circulating biomarkers and HF severity, as assessed by NYHA cardiac function classification. Notably, patients with higher NYHA functional classes exhibited elevated serum NT-proBNP and plasma TGF-β1 levels, indicating their potential prognostic significance in gauging HF severity and progression in T2DM patients. These observations are consistent with prior studies demonstrating the prognostic value of NT-proBNP and TGF-β1 in predicting adverse cardiovascular outcomes and mortality in HF patients [13] [14].

Cardiomyopathy, acute and chronic myocardial infarction and other heart-related diseases can cause myocardial injury, inducing abnormal changes in the structure and function of the myocardium, which reduces the contractile function of the patient’s heart, decreases the filling function of the ventricle, and ultimately causes the occurrence of chronic HF, leading to severe threats to the patient’s physical health [15] [16] [17]. In addition to direct cardiac disease triggering,T2DM is also an important factor in damaging myocardial function to trigger HF. The incidence of HF in diabetic patients is about 9% to 22% [18] [19] [20]. In clinical studies in relevant animal models, diabetes causes myocardial steatosis, increasing the thickness of the left ventricular wall, which in turn induces centripetal remodelling of the left ventricle. This process is one of the physiological processes contributing to the development of HF [21] [22].

Our study found that NT-proBNP levels were significantly higher in T2DM patients with HF than those without. This is consistent with other studies. For instance, a study published in the American College of Cardiology found that NT-proBNP levels increase significantly in HF patients [23]. Another study found that each doubling of baseline NT-proBNP was associated with a hazard ratio of 1.17 for CV death or HF hospitalisation [24].

This study showed that TGF-β1 levels were significantly higher in T2DM patients with HF. While limited studies specifically investigate TGF-β1 levels in T2DM patients with HF, TGF-β1 has been studied in other contexts. For example, a study found that serum TGF-β1 levels were significantly lower in patients with coronary artery ectasia than in controls [25]. TGF-β1 had a good predictive efficacy for diagnosing concurrent HF in patients with T2DM, with an AUC of 0.803. This is similar to a study on coronary artery ectasia patients, where the AUC value of serum TGF-β1 levels for predicting CAE was 0.64 [25].

TGF-β1 produces reactive oxygen species and the inflammatory response through autocrine pathways, among other physiological processes. Even though all three of TGF-’s isoforms in the human body share many biological similarities, TGF-β1 is the most active and contributes the most to controlling different cell physiological activities [26] [27]. TGF-β1 can play a role in the beginning and progression of fibrosis in diabetic nephropathy by causing epithelial mesangialization of renal tissue, which in turn causes glomerular mesangial fibrosis, according to previous clinical research [28] [29]. In addition to diabetic nephropathy, cardiovascular complications also have a high incidence in diabetic patients, mainly due to metabolic function abnormalities leading to impaired myocardial function in diabetic patients. Qin Chaoshi et al. [30] showed that TGF-β1 overexpression could activate myocardial oxidative stress and inflammatory response, increase cardiomyocyte apoptosis, and induce myocardial injury in a T2DM cardiomyopathy mouse model. As a result, the current study concluded that TGF-β1 expression is crucial in T2DM with HF.

In this study, among 150 T2DM patients who visited the clinic for dyspnoea, the detection rate of HF was 64.0% (96/150), which shows that HF is more common in T2DM patients, but it is difficult to accurately assess the occurrence of HF solely based on the symptom of dyspnea. Serum TGF-β1 levels in the current study’s T2DM patients were higher than those in healthy individuals who did not have diabetes or cardiac impairment, indicating that TGF-β1 is more active in T2DM patients. The current study’s T2DM patients had serum TGF-β1 levels that were higher than those of healthy people who did not have diabetes or cardiac impairment, showing that TGF-β1 is more active in T2DM patients. The sensitivity and specificity for diagnosing concomitant HF in patients with T2DM were 79.63%, respectively, and 52.51%. It is suggested that plasma TGF-β1 level has the application value of predicting HF in T2DM patients.

A strong positive correlation (r=0.784, p<0.0001) between plasma TGF-β1 and serum NT-proBNP levels in patients with T2DM and HF. While no specific studies investigate the correlation between NT-proBNP and TGF-β1 in T2DM patients with HF, both markers have been independently associated with HF in various studies [31] [32] [33].

In conclusion, plasma TGF-β1 level has some diagnostic use in T2DM with HF, and patients’ plasma TGF-β1 level steadily rises as they experience HF symptoms. Deficiencies in this study: Throughout the course of the study, it was discovered that this index is not only involved in the development of the disease but also in the occurrence of HF. It is, therefore, implied that the reformulation of the representative has the same effect of predicting the prognosis of patients with T2DM with HF.

## Dodatak

### Prognostic effect

However, since the patients were not followed up in this study, verifying the reformulation conjecture was impossible.

### Suggestion for improvement

It is suggested that follow-up studies could be conducted for patients with T2DM with HF diagnosed for the first time and verify the value of plasma TGF-β1 levels in the prognosis of patients with T2DM with HF.

### Conflict of interest statement

All the authors declare that they have no conflict of interest in this work.
